# Predictors of Self-Management Behaviors in Older Adults with Hypertension

**DOI:** 10.1155/2015/960263

**Published:** 2015-08-12

**Authors:** Brenda M. Douglas, Elizabeth P. Howard

**Affiliations:** Northeastern University, 360 Huntington Avenue, 103 Robinson Hall, Boston, MA 02115, USA

## Abstract

The purpose of this study was to develop a prediction model of demographic and sociobehavioral characteristics common among older adults with hypertension (HTN) who engage in self-management behavior. A descriptive, correlational predictive design was used to collect data at 14 faith-based and senior citizen organizations in a major urban northeastern city. Participants ranged in age from 63 to 96 with a mean age of 77 (SD 6.9). A 33-item questionnaire was used to gather data on 15 explanatory and 5 outcome variables. Instruments were the Perceived Stress Scale, the Duke Social Support Index, the stage of change for physical activity scale, and the DASH Food Frequency Questionnaire. Correlation and regression analyses were used to test the hypothesis. Results indicate there is a common set of characteristics such as higher stage of change, reading food labels, and higher self-rated health that can predict the older adult's likelihood to engage in hypertension self-management behavior. The significant correlations found in this preliminary study warrant further study and validation. Findings are clinically relevant as knowledge of demographic and sociobehavioral characteristics associated with engagement in self-management behavior enables health care clinicians to support and encourage older adults to improve management of this common, chronic condition.

## 1. Introduction

In the US, hypertension (HTN) is ranked as the most common primary diagnosis for which adults seek medical attention [1]. Affecting 1 in 3 adults, its prevalence rises across all age-specific categories, particularly those aged 60 and older [2]. Data from the 2005-2006 National Health and Nutrition Examination Survey (NHANES) show that 67% of adults aged 60 and older have high blood pressure compared to 30% for those aged 40 to 59 [3]. Findings from the Framingham Heart Study have demonstrated those who are normotensive at age 65 have a 90% lifetime risk of developing hypertension by age 85 [4].

While the burden of HTN among older adults is increasing, the ability to control HTN has remained well below the Healthy People 2020 goal of 50%. Based on analysis of 2005–2008 NHANES data, the control rate for all adults is 43.7% [5]. The challenge of improving HTN control is significant particularly given the relationship between uncontrolled HTN and cardiovascular disease (CVD), heart failure, stroke, kidney disease, and retinal diseases. The burden for health care clinicians is to identify and intervene appropriately to encourage and support engagement in hypertension self-management behaviors to achieve control and mitigate the development of complications. A prediction model of those likely to engage in self-management behavior can target at-risk individuals and guide health care interactions.

## 2. Background

Engagement in self-management behavior is indispensable for the management of HTN per the Eighth Joint National Committee for the Prevention, Detection, Evaluation, and Treatment of High Blood Pressure (JNC-8) [1]. One Healthy People 2020 objective is to increase the proportion of prehypertensive and hypertensive adults who meet the recommended guidelines for body mass index (BMI), saturated fat consumption, sodium intake, physical activity, and alcohol consumption [6]. This objective supports the importance of self-management behaviors for HTN prevention and management across all stages and mirrors lifestyle change recommendations by the JNC-8.

## 3. Self-Management Behaviors

Hypertension self-management behaviors recommended by the JNC-8 are weight reduction in overweight or obese individuals, adoption of the Dietary Approaches to Stop Hypertension (DASH) diet, adoption of a low sodium diet, engagement in physical activity, and moderation in alcohol consumption [1]. There is strong and consistent evidence linking high body mass index (BMI), unhealthy dietary practices, high sodium intake, decreased physical activity, and high alcohol consumption to the incidence of HTN [7]. Overwhelming, substantive evidence exists on the significant effects of self-management behavior on blood pressure [1, 8]. Studies demonstrate it is possible for adults, including older adults, to achieve lower blood pressure by adopting self-management behaviors. For effective, nonpharmacological management of HTN, the challenge begins with determining those most likely to engage in self-management behaviors.

A primary objective in the evaluation of individuals with HTN is to assess adherence to self-management behaviors to help guide treatment decisions [1]. However, even with the known association between the performance of self-management behavior and lower blood pressure levels, inadequate or inconsistent attention is given to lifestyle change during routine health care encounters [9, 10]. Performance evaluation of self-management activities and the subsequent provision of advice or education to promote consistent adoption to manage HTN is not a routine part of HTN management. Suboptimal care delivery to patients with HTN is evident in the inconsistency that exists between self-management behavior guideline recommendations and what occurs during the patient-clinician encounters.

A prediction model of demographic and sociobehavioral characteristics common among older adults with HTN who engage in self-management behavior may guide clinician dialogue to encourage engagement or support sustainability in self-management behaviors. This project aimed to test the hypothesis that there is a common set of characteristics that can predict the older hypertensive adults who are likely to engage in self-management behavior.

## 4. Conceptual Framework

The goal of the Interaction Model of Client Health Behavior (IMCHB) is to identify explanatory relationships to facilitate patient care interventions, a goal consistent with the aim of this study. The background elements in this model are inclusive of demographic and sociobehavioral characteristics and are recognized as antecedents to health-related outcomes, defined in this study as engagement in self-management behavior. Explanatory variables in the study were selected based on empiric and theoretical support that the characteristic was associated with at least one self-management behavior.

## 5. Settings and Sample

Data were collected at 14 faith-based and senior citizen organizations in a major urban northeastern city. A total of 166 questionnaires were hand distributed and collected; however, 10 were incomplete and therefore excluded from the analysis. The final number of study participants was 156.

Participants ranged in age from 63 to 96 with a mean age of 77 (SD 6.9). The largest age group was represented by those 70–79 years of age (*n* = 74, 47.4%) followed by the oldest participants between the ages of 80–96 (*n* = 55, 35.3%). Gender was unevenly represented with 80.8% (*n* = 126) women although reflective of the population. Eighty-four participants were white (53.8%), and 67 were African American or black (42.9%). The remainder represented other ethnic and racial groups. More participants were widowed (*n* = 76, 48.7%) than were married (*n* = 40, 25.6%). Among other categories, divorced participants were approximately equal to those who never married, *n* = 18 (11.5%) and *n* = 19 (12.2%), respectively, and 3 (1.9%) participants were separated. Approximately 90% of participants completed education at a high school or higher level. Seventy participants (44.9%) completed grade 12 or earned a GED, 38 (24.4%) completed 1–3 years of college, and 31 (19.9%) completed 4 or more years of college.

The majority of participants (84%) reported having only HTN, or HTN and one other comorbidity. The most commonly cited comorbidities, cancer (5.8%) and diabetes (12.2%), were not surprising given the lifetime cancer prevalence rate is 41.6% for men and 32.2% for women, and the prevalence rate for diabetes among those aged 65 and older is 26.9% [11, 12].

## 6. Data Collection

Permission to conduct the study was received through a University Institutional Review Board. The researchers compiled a 33-item questionnaire to gather data on 15 explanatory variables and 5 outcome variables. Three of the explanatory variables and one outcome variable were measured using valid and reliable instruments: perceived stress was measured using the Perceived Stress Scale (PSS4), social support was measured using the Duke Social Support Index (DSSI), stage of change (SOC) was measured using the SOC for physical activity, and adherence to the DASH diet plan was measured using the DASH Food Frequency Questionnaire (DASH FFQ). The remaining explanatory and outcome variables were adapted from the Behavioral Risk Factor Surveillance Survey (BRFSS), the Inter-RAI Community Health Assessment (CHA), and the NHANES 2007-2008 and from a previous study focused on primary care management of HTN in older adults [13–16].

## 7. Results

Regression analyses were conducted to determine if the independent variables, demographic and sociobehavioral characteristics, were predictive of the five self-management behaviors. Multiple linear regression was used for continuous outcome variables, and logistic regression was used for dichotomous outcome variables. The stepwise method was used for entering explanatory variables into the regression equation. Finally, each outcome variable was tested with the predictor variables to determine the best set of predictors.

To assess correlations among the predictors, the Pearson product moment correlation coefficient was used for variables measured at a ratio level of measurement: age, the PSS score, the DSSI score, and the number of comorbidities. The remaining predictor variables were either dichotomous or an ordinal scale and Kendall's Tau-*b* test was used. A two-tailed test for significance was done since the direction was not predicted. Review of the correlation coefficients and the level of significance revealed that although there were correlations that reached significance (*P* < 0.05), the highest correlation was between marital status and gender with a correlation coefficient of 0.260 (*P* = 0.000). Since the coefficients were less than 0.70, the 15 explanatory variables were retained for entry into regression analyses.

Multiple linear regression using the stepwise method determined the explanatory variables predictive of BMI. The model (Table 1) shows SOC (*P* < 0.000) and diabetes (*P* < 0.012) as significant predictors of BMI. The beta standardized coefficients show a negative relationship between SOC and BMI indicating that those at a higher SOC have a lower BMI. Diabetes had a positive relationship to BMI such that having diabetes is associated with having a higher BMI. The adjusted *R*-squared indicates that SOC and diabetes accounted for 13.3% (*P* = 0.000) of the variance of BMI.

The total DASH score was calculated from responses to 8 questions with a score range of 19–55. As a continuous variable, multiple linear regression using the stepwise method determined the explanatory variables predictive of following a DASH diet plan. When DASH was regressed on three predictor variables, SOC, age, and self-rated health accounted for 15% of the variance in DASH adherence. The final model was significant at *P* = 0.000 level.  Table 2 shows the significant contribution of each predictor to the variance in DASH. Results indicate that being separated (marital status), reading food labels, and having more education are associated with having a higher DASH score.

Two regression models were tested to determine predictors of regular exercise. The exercise variable was dichotomized to those who exercise (2-3, 4-5, or more than 4 hours in the last 3 days), and those who do not exercise (none, less than 1 hour, or 1-2 hours in the past 3 days). When exercise was used as a dichotomous variable, higher self-rated health was revealed as a significant predictor of those in the exercise group. However, when exercise as an interval measure was entered into the model, SOC and age were also significant predictors (*P* < 0.05) as shown in Table 3.

There is a negative relationship between age and exercise, and a positive relationship between exercise and SOC and self-rated health indicating that being younger, being at a higher SOC, and having higher self-rated health are associated with more exercise (*R* ^2^ = 15.2, *P* = 0.000).

Responses to adherence to a low sodium diet were dichotomized with “quite a bit” and “very much” in one category and “not at all,” “a little,” and “somewhat” in the other. Explanatory variables significant to the model were age (*P* = 0.05) and reading food labels (*P* = 0.001) as indicated in Table 4. Increasing age and reading food labels either “quite a bit” or “very much” increased odds of consuming a low sodium diet.

Because data collected for alcohol intake were highly skewed, responses were categorized into recommended versus not recommended consumption quantities: having 0-1 drink per day for women and having 0–2 drinks per day for men were categorized as one group, and drinking more per day for either gender was classified as not recommended consumption. Regression analysis was used to determine which factors affect the probability of consuming alcohol in recommended quantities. As summarized in Table 5, increasing age, having diabetes, and gender (female) were associated with consuming the recommended amount of alcohol.

## 8. Discussion

This project aimed to develop a prediction model of demographic and sociobehavioral characteristics found in older adults with HTN who engage in self-management behavior. There is support for the hypothesis that a common set of characteristics predict the older hypertensive adult who is likely to engage in self-management behaviors, and a select group of characteristics are associated with each self-management behavior. The significant factors for each self-management behavior are depicted in Table 6.

Being in the “active” or “maintenance” SOC is a significant predictor of a normal BMI, and being in the “active” or “maintenance” SOC and higher self-rated health are significantly associated with participation in regular exercise. Self-efficacy, as a mediating influence, has been found to link these variables in other studies. Resnick and Nigg found that the influence of positive self-rated health on self-efficacy directly influenced SOC and engagement in exercise [17]. Similarly, a randomized controlled single blind study of community dwelling older adults with chronic disease aged 70–92 found a positive association between self-rated health and exercise, as did a study of community dwelling older adults using accelerometers as an objective measure of physical activity [18, 19].

Having diabetes as a significant predictor of a normal BMI is not supported by NHANES III data on adults with Type 2 diabetes that revealed 54.8% were obese (BMI = ≥30 kg/m^2^), 31% reported no leisure time activity, and 38% did not meet physical activity recommendations [20]. The high level of health consciousness and health knowledge of the current study group may have impacted this finding given that physical activity can improve glycemic control and insulin resistance. The association between younger age and exercise may speak to the likelihood that as people enter their 8th and 9th decade, they experience comorbidities that impact their ability to remain physically active.

Adherence to the DASH diet and following a low sodium diet were each associated with reading food labels, and adhering to a low sodium diet was additionally associated with older age. There is evidence that older adults who are health conscious and knowledgeable about health promotion are more likely to read food labels when making purchasing decisions. A study of correlates of food label use among older adults found that, for both males and females, having good diet quality perception was a significant predictor of food label use [21]. Implementing a telephone survey with a national probability sample of 475 older adults, Elbon et al. found that being female, having a high level of nutrition knowledge, and positive health seeking behaviors were associated with reading food labels [22]. In another study, older age and being female were significant predictors of purchasing healthy food items such that a 10-year increase in age found a 29% increase in the likelihood of purchasing a healthy food item [23]. Education additionally was found to be predictive of adhering to a DASH diet. Despite this factor as well as the “separated” marital status being found predictive of adhering to a DASH diet in the current study, there is no research to support these outcomes.

Predictors of consuming alcohol use within recommended amounts were not having a diagnosis of diabetes, younger age, and being female. A longitudinal study over 20 years in a southern California community provides partial support for these findings. The study found that although older adults consumed less alcohol as they aged, more males than females—35% and 24%, respectively—continued to consume higher amounts than recommended guidelines, a finding that held regardless of having a comorbid chronic illness such as HTN (28%) and diabetes (31%) [24].

Examination of the sociobehavioral characteristics of the study population revealed a relatively healthy group of older adults with HTN. This is supported by the 74.4% that self-assessed their health as “good” or “excellent.” This outcome is similar to data collected in the 2009 National Health Interview Survey where 76% of individuals aged 65 and older reported their health to be “good” or “excellent” [12]. In the current study, not only were study participants optimistic about their health, but they were vested in the health maintenance and desired to be actively engaged in managing their health outcomes, a finding consistent with other studies of older adults [25, 26]. The optimism older adults have to achieve healthy aging has been associated with engagement in health-promoting behaviors [27].

It is surprising that, given the health consciousness of the study population, less than 25% practiced home blood pressure monitoring (HBPM), a result that differs dramatically from a cross-sectional study of 150 hypertensive adults where 63% reported consistently measuring blood pressure at home [28]. A possible explanation is that the study population may have had access to regular health care and free blood pressure screening clinics tempering the need for home monitoring. Regardless, HBPM is well supported by visible organizations such as the American Heart Association and is becoming a recognized standard part of HTN self-management [29].

BMI was relatively evenly distributed between those who were normal weight, overweight, and obese. It is notable that 64.1% of the participants were either overweight or obese, a proportion similar to population estimates. Data from the 2007-2008 NHANES survey compared with data from 1999 through 2006 found that the combined prevalence for overweight and obesity for individuals aged 60 and older (BMI > 25 kg/m^2^) was 78.4% for men (95% CI 74.8–81.9%), and 68.6% for women (95% CI 644–72.7%) [30]. Current understanding of the “obesity paradox” recognizes that additional weight may be protective in older adults and decrease the mortality risk in those with HTN and other diseases which is encouraging in light of the study finding [31]. The challenge is in preserving lean body mass and muscle strength while managing body fat.

## 9. Limitations

The use of a nonprobability convenience sampling method is a recognized limitation. The homogeneity of the participants was another limitation. The similarity among the participants in the attributes being studied contributed to less variability among responses and potentially limited explanatory variables from reaching a level of significance. Seven of the 12 data collection sites were faith-based organizations, while 5 were city-based senior citizen organizations. Recruiting from a more diverse set of venues, including senior residential communities, independent living facilities, adult day health care centers, and senior social and activity groups, would likely increase variability among responses and increase sample representativeness. Particular consideration should be given to venues where older men are likely to be recruited. The concept of social desirability is another potential limitation. Questionnaires were completed with participants sitting next to each other and the desire to be viewed favorably by a fellow participant may have prompted socially acceptable responses.

## 10. Conclusions

The study found that select demographic and sociobehavioral characteristics are related to engagement in self-management behaviors. Regression analyses identified each significant correlation to be a significant predictor of one or more self-management behaviors. While the findings of the current study were not robust, they are significant in identifying a common set of demographic and sociobehavioral characteristics predictive of engagement in self-management behaviors that may be used to direct further research. A positive assessment of the study population that has implications for practice is that older adults are actively engaged in and knowledgeable about self-management behaviors to manage the pervasive problem of HTN.

## Figures and Tables

**Table 1:** Predictors of BMI.

Model	Unstandardized coefficients		Standardized coefficients	*t*	Sig.
	*B*	Std. error	Beta		
1					
(Constant)	31.452	.958		32.845	.000
SOC	−1.309	.302	−.330	−4.335	.000
2					
(Constant)	31.059	.954		32.558	.000
SOC	−1.309	.297	−.330	−4.407	.000
Diabetes	3.207	1.268	.189	2.529	.012

Dependent variable: BMI.

**Table 2:** Predictors of a DASH diet plan.

Model	Unstandardized coefficients		Standardized coefficients	*t*	Sig.
	*B*	Std. error	Beta		
1					
(Constant)	32.480	.495		65.661	.000
Separated	11.853	3.556	.260	3.334	.001
2					
(Constant)	29.719	1.028		28.902	.000
Separated	10.708	3.484	.235	3.074	.003
Food labels 0–5	1.065	.350	.232	3.040	.003
3					
(Constant)	25.167	1.878		13.398	.000
Separated	10.495	3.405	.230	3.082	.002
Food labels 0–5	1.043	.342	.228	3.045	.003
Education	1.322	.461	.213	2.868	.005

Dependent variable: DASH.

**Table 3:** Predictors of exercise.

Model	Unstandardized coefficients		Standardized coefficients	*t*	Sig.
	*B*	Std. error	Beta		
1					
(Constant)	1.394	.259		5.376	.000
SOC	.332	.082	.311	4.065	.000
2					
(Constant)	4.924	1.253		3.929	.000
SOC	.364	.081	.341	4.511	.000
Age	−.047	.016	−.217	−2.876	.005
3					
(Constant)	3.949	1.318		2.996	.003
SOC	.343	.080	.321	4.270	.000
Age	−.049	.016	−.226	−3.026	.003
Self-rated health	.416	.192	.162	2.163	.032

Dependent variable: exercises 0–5.

**Table 4:** Predictors of low sodium diet.

Coefficients	Estimate	Std. error	*z* value	Pr. (>|*z*|)	*P* value
(Intercept)	−5.14766	2.07568	−2.480	0.0131	0.001
Age	0.07617	0.02842	2.680	0.007369	0.05
Food labels	1.75985	0.38646	4.554	5.27*e* − 06	0.001

**Table 5:** Predictors of alcohol use.

Coefficients	Estimate	Std. error	*z* value	Pr. (>|*z*|)	*P* value
(Intercept)	2.06909	2.37050	0.873	0.3827	
Age	−0.06664	0.03087	−2.159	0.0309	0.05
Gender	1.40426	0.67035	2.095	0.0362	0.05
Diabetes	−2.24293	1.06975	−2.097	0.0360	0.05

**Table 6:** Final predictors of engagement in TLC.

BMI	DASH	Exercise	Low Na^+^ diet	Alcohol use
SOC .000		SOC .000		
Diabetes .012				Diabetes .05
	Separated .002			
	Read labels .003		Read labels .001	
	Education .005			
		Age .003	Age .05	Age .05
		Self-rated health .032		
				Gender .05
